# High-Frequency Repetitive Transcranial Magnetic Stimulation Mediates Autophagy Flux in Human Bone Mesenchymal Stromal Cells via NMDA Receptor–Ca^2+^–Extracellular Signal-Regulated Kinase–Mammalian Target of Rapamycin Signaling

**DOI:** 10.3389/fnins.2019.01225

**Published:** 2019-11-19

**Authors:** Xinlong Wang, Xing Zhou, Jie Bao, Zhiguo Chen, Jingzhao Tang, Xueyang Gong, Jing Ni, Qi Fang, Yaobo Liu, Min Su

**Affiliations:** ^1^Department of Physical Medicine and Rehabilitation, The First Affiliated Hospital of Soochow University, Suzhou, China; ^2^Department of Children’s Health Care Center, Wuxi Children’s Hospital, Wuxi, China; ^3^Sport Rehabilitation Center of Physical and Education School, Soochow University, Suzhou, China; ^4^Department of Neurology, The First Affiliated Hospital of Soochow University, Suzhou, China; ^5^Department of Medical Rehabilitation, Community Health Service Center of Yangming Street, Wuxi, China; ^6^Department of Cardiopulmonary Rehabilitation, Wuxi Tongren Rehabilitation Hospital, Wuxi, China; ^7^Department of Geriatric Rehabilitation, Jiangsu Rongjun Hospital, Wuxi, China; ^8^Jiangsu Key Laboratory of Neuropsychiatric Diseases and Institute of Neuroscience, Soochow University, Suzhou, China

**Keywords:** repetitive transcranial magnetic stimulation, autophagy, bone mesenchymal stromal cells, extracellular signal-regulated kinase–mammalian target of rapamycin signaling, NMDA receptor

## Abstract

**Aim:**

Repetitive transcranial magnetic stimulation (rTMS) is a non-invasive and safe technique for treatment of central and peripheral nerve injury. In recent years, this technique has been widely used in clinic, and an increasing number of studies have reported its mechanisms. In this study, we investigated the mechanisms of rTMS-mediated autophagy flux in human bone mesenchymal stromal cells (BMSCs).

**Methods:**

A frequency of 50 Hz was employed. Cells were divided into five groups: (1) normal, (2) sham, (3) 0.5 T, (4) 1.0 T, and (5) 1.5 T. Cells were stimulated for 20 min/day. The levels of p62, LC3-II/I, phosphorylated extracellular signal-regulated kinase (p-ERK), ERK, phosphorylated-AKT (p-AKT), AKT, phosphorylated mammalian target of rapamycin (p-mTOR), mTOR, phosphorylated protein kinase A (p-PKA), PKA, phosphorylated epidermal growth factor receptor (p-EGFR), EGFR, Nanog, Oct4, Sox2, and NMDA receptor (NMDAR1) were investigated by western blotting. Intracellular calcium (Ca^2+^) levels were quantified by flow cytometry. p62 and LC3 expression was also assessed by immunofluorescence analysis.

**Results:**

In the 0.5 T group, rTMS increased the expression of LC3-II/I, p-ERK/ERK, and NMDAR1 and decreased the levels of p62 and p-mTOR/mTOR than in the normal group. The ratio of p-AKT/AKT, p-PKA/PKA, and p-EGFR/EGFR and the expression of Nanog, Oct4, and Sox2 remained unchanged. Immunofluorescence analysis revealed colocalization of p62 with LC3 puncta, and flow cytometry analysis displayed that Ca^2+^ levels were elevated. However, in the 1.0 and 1.5 T groups, no changes in the expression of these autophagy markers were observed.

**Conclusion:**

In the 0.5 T group, high-frequency rTMS can induce autophagy through NMDAR–Ca^2+^–ERK–mTOR signaling in BMSCs. In the 1.0 and 1.5 T groups, autophagy is not activated.

## Introduction

Repetitive transcranial magnetic stimulation (rTMS) is widely used for the treatment of central nervous system (CNS) diseases and is based on the regulation of brain plasticity and electromagnetic induction (Matheson et al., 2016). The effects of rTMS are determined by various stimulatory parameters including stimulus intensity, stimulus frequency, and stimulus patterns. Frequency remains a crucial factor. High-frequency rTMS (≥5 Hz) can facilitate cortical excitability, whereas low-frequency rTMS (≤1 Hz) reduces cortical excitability (Shu et al., 2018). Previous studies found that rTMS could treat CNS disease, such as Parkinson’s disease and Alzheimer’s disease (Rabey and Dobronevsky, 2016; Yang et al., 2018). In addition, rTMS can intervene in various electrostatic processes, such as protein–protein interactions (To et al., 2018). However, the molecular mechanisms of these effects are not completely understood.

Stem cell therapy is an effective, safe, and promising treatment option for neurological disease. Although neural stem cells are a useful treatment modality for CNS diseases, the heterogeneity of their differentiation in various neurospheres limit their utility. Compared with neural stem cells, BMSCs are rich and easy to cultivate, and auto-transplantation can be performed. BMSCs can differentiate into neuron-like cells and glial cells, which offers a potential option for CNS diseases such as Parkinson’s and Alzheimer’s (Abe et al., 2012). It has been reported that cotherapy with rTMS and BMSCs produces therapeutic benefits in vascular dementia rat models (Wang et al., 2018). Whether these therapies can be combined to treat other complex diseases, such as Parkinson’s and Alzheimer’s diseases, has not been investigated. To study this potential, we must first understand the mechanisms underlying the effects of rTMS on BMSCs.

Autophagy is formed by double-membraned autophagosomes that eliminate abnormal protein aggregates and damaged organelles. Autophagosomes fuse with lysosomes to degrade cellular materials. Two commonly used autophagy markers are LC3-II/I and p62/SQSTM1. LC3 is cleaved at its C-terminus by ATG4 to become a cytoplasmic soluble form of LC3-I. LC3-I is then lipidated to form LC3-II, which can remain on autophagosomes until their fusion with lysosomes. The ratio of LC3-II/I is considered an accurate indicator of autophagosome formation (Wu et al., 2016). p62 is an adaptor protein that correlates with autophagic degradation. The inhibition of autophagy increases the levels of p62, whereas decreases levels lead to autophagy activation. Therefore, p62 is a commonly used marker to assess autophagic flux (Lipinski et al., 2015; Li et al., 2016). The dysfunction of autophagy leads to neurodegeneration and cell death (Zhang et al., 2017). For this reason, autophagy has become an important therapeutic target for neurological diseases, and various clinical interventions can enhance autophagy (Towers and Thorburn, 2016). In Parkinson’s disease, autophagy promotes the clearance of α-synuclein, a nuclein that is prone to aggregation and has emerged as a therapeutic target to delay disease progression (Su et al., 2015). For Alzheimer’s disease, autophagy clears tau protein aggregates and reduces neuronal cytotoxicity (Fang et al., 2019). Autophagy also has protective effects on peri-ischemic brain tissue in ischemic rat models (Ao et al., 2019).

In this study, we investigated the link among rTMS, BMSC, and autophagy. We further explored the molecular mechanisms of the rTMS regulation of BMSCs.

## Materials and Methods

### Materials and Apparatus

Repetitive transcranial magnetic stimulation was purchased from Yingzhi Company (M-100 Ultimate). All reagents are listed as follows: phosphorylated-AKT (p-AKT) (4060, Cell Signaling), AKT (4691, Cell Signaling), phosphorylated extracellular signal-regulated kinase (p-ERK) (50011, Abcam), ERK (184699, Abcam), phosphorylated epidermal growth factor receptor (p-EGFR) (40815, Abcam), EGFR (52894, Abcam), NMDA receptor (NMDAR1) (17345, Abcam), p62 (56416, Abcam), LC3 (192890, Abcam), phosphorylated mammalian target of rapamycin (p-mTOR) (5536, Cell Signaling), mTOR (2983, Cell Signaling), phosphorylated protein kinase A (p-PKA) (32390, Abcam), PKA (75993, Abcam), Oct4 (2750, Cell Signaling), Nanog (8822, Cell Signaling), Sox2 (3579, Cell Signaling), GAPDH (8245, Abcam), dizocilpine maleate (MK801) (15084, MCE), 1,4-diamino-2,3-dicyano-1,4-bis(*o*-aminophenylmercapto)butadiene (U0126) (1901, Beyotime), 3-benzyl-5-((2-nitrophenoxy)methyl)-dihydrofuran-2(3*H*)-one (3BDO) (8317, Selleck), Fluo-4/AM (1060, Beyotime), horseradish peroxidase (HRP)-mouse (931, Sigma), HRP-rabbit (934, Sigma), Alexa Fluor 488 (150077, Abcam), Alexa Fluor Cy3 (97035, Abcam), and Cell Counting Kit-8 (CCK-8) (0037, Beyotime).

### Cell Culture

Human bone mesenchymal stromal cells (BMSCs) were purchased from Cyagen Biosciences Inc. (HUXMA-01201). Cells were maintained in Dulbecco’s modified Eagle’s medium (11995, Gibco) and supplemented with 10% fetal bovine serum (FBS) (11995, Gibco) containing 1% penicillin/streptomycin, 0.2% herin, and 1% glutamax at 37°C in a humidified atmosphere containing 5% CO_2_. Cells were pretreated with inhibitors for 1 h prior to stimulation.

### Repetitive Transcranial Magnetic Stimulation

**FIGURE 1:**
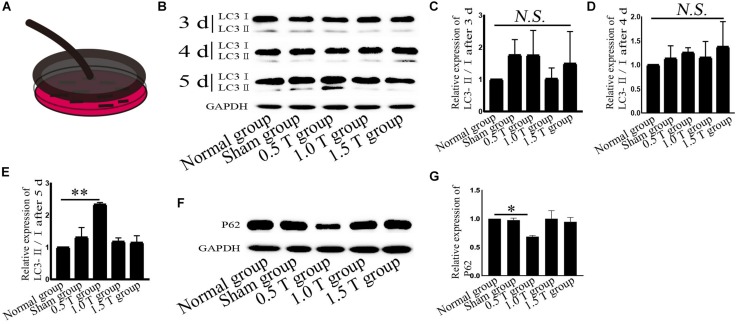
Repetitive transcranial magnetic stimulation (rTMS) induces autophagy. **(A)** Model of rTMS stimulation of bone mesenchymal stromal cells (BMSCs). **(B)** The autophagy related-protein LC3 was assessed by western blotting. **(C–E)** Quantification of LC3-II/I. *n* = 3. Data were analyzed with a one-way ANOVA followed by Dunnett’s multiple comparison test. NS, not significant, ^∗∗^*P* < 0.01. Error bars = SD. The LC3-II/I ratio after 5 days of 0.5 T was 2.340 ± 0.057. **(F)** Autophagy related-p62 expression assessed by western blotting. **(G)** Quantification of western blotting for p62. *n* = 3. Data were analyzed with a one-way ANOVA followed by Dunnett’s multiple comparison test. ^∗^*P* < 0.05. Error bars = SD. The value of p62 after 5 days of 0.5 T was 0.685 ± 0.021.

The rTMS equipment was connected to a standard 70-mm outer wing diameter double that was a figure-of-eight coil with a frequency of 50 Hz. And the equipment rTMS had three magnetic strengths: 0.5, 1.0, and 1.5 T. Each dish has 2 × 10^5^ cells. BMSCs were randomly divided into five groups: (1) normal, (2) sham, (3) 0.5 T, (4) 1.0 T, and (5) 1.5 T groups. Dishes were placed below the coil, and the distance between the coil and culture dish was 1.0 cm. The stimulation consisted of 20 trains of 100 pulses delivered at 50 Hz (2 s each train) with an inter-train interval of 58 s to allow effective cooling of the coil. rTMS was applied for 20 min once a day for three, four, and five consecutive days. For the 0.5 T group, 1.0 T group, and 1.5 T group, the stimulation intensities were 0.5, 1.0, and 1.5 T, respectively. The normal group did not receive treatments. Cells in the sham group were exposed to the device but were not actively stimulated (Figure 1A).

### Western Blotting

Samples were mechanically dissociated and lysed in radio-immunoprecipitation assay (RIPA) buffer (50 mM of Tris–HCl, 150 mM of NaCl, 1 mM of Na_2_-EDTA, 1% NP-40, and 0.25% Na-deoxycholate) containing protease inhibitor cocktail (04693132001, Roche) and phosphatase inhibitor cocktail (04906845001, Roche). After pretreatment, proteins were subjected to sodium dodecyl sulfate–polyacrylamide gel electrophoresis (SDS-PAGE) and transferred to polyvinylidene difluoride (PVDF) membranes. Blots were incubated at room temperature in blocking buffer containing 5% skimmed milk for 1 h and probed with primary antibodies (p-AKT, 1:2,000; AKT, 1:2,000; p-ERK, 1:2,000; ERK, 1:2,000; p-EGFR, 1:2,000; EGFR, 1:2,000; p-PKA, 1:2,000; PKA, 1:2,000; NMDAR1, 1:2,000; p62, 1:2,000; LC3-I/II, 1:2,000; p-mTOR, 1:2,000; mTOR, 1:2,000; Nanog, 1:2,000; Oct4, 1:2,000; Sox2, 1:2,000; and GAPDH, 1:5,000) overnight at 4°C. Blots were labeled with secondary antibodies for 1 h at room temperature. Blots were developed with enhanced chemiluminescence (ECL) and visualized using ImageLab^TM^ software. Band intensities were obtained using ImageJ (National Institutes of Health [NIH]) software.

### Cell Viability

Cell viability assays were performed with CCK-8 assays. Briefly, cells (∼2 × 10^3^ cells) were treated with rTMS for 5 days, and 10 μl of CCK-8 was added to each well for 1.5 h. Absorbances were measured at 450 nm using a microplate reader (Tecan M200, Grodig, Austria).

### Detection of Intracellular Ca^2+^ Concentrations

Ca^2+^ signals were measured with Fluo-4/AM (a cytosolic Ca^2+^ indicator) according to the manufacturers’ instructions. Briefly, cells were digested with 0.25% trypsin and stained with 200 μl of Fluo-4/AM (5 μmol/L) at 37°C for 30 min. Fluorescence intensities were detected by flow cytometry (Beckman Coulter, United States).

### Immunofluorescence

Cells were fixed with absolute methanol for 5 min, permeabilized with 0.1% Triton X-100 for 5 min, and blocked in 0.1% phosphate-buffered saline (PBS)–Tween containing 1% bovine serum albumin (BSA), 10% FBS, and 0.3 M of glycine for 1 h. Cells were probed with primary antibodies (LC3-I/II, 1:300; P62, 1:300) overnight at 4°C and stained with secondary antibodies (Alexa Cy3-conjugated anti-mouse IgG, 1:500; Alexa 488-conjugated anti-rabbit IgG, 1:500) at room temperature for 1 h. Cells were observed under a Zeiss LSM700 confocal microscope and quantified by ImageJ (NIH). From each group, a minimum of 60 cells were analyzed.

### Statistical Analysis

Data are shown as the mean ± SD using GraphPad Prism 8.01. A Student’s *t*-test was used for single comparisons between the two groups. Other data were analyzed using a one-way ANOVA followed by Dunnett’s multiple comparison test. ^∗^*P* < 0.05, ^∗∗^*P* < 0.01, ^∗∗∗^*P* < 0.001, or no significant difference (NS) denote the significance thresholds.

## Results

### Repetitive Transcranial Magnetic Stimulation Promotes Autophagic Flux

**FIGURE 2:**
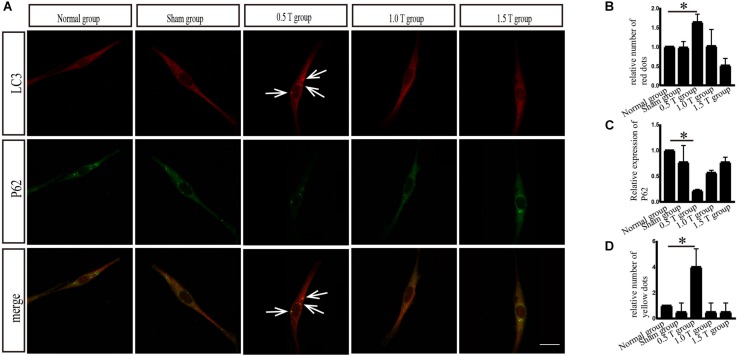
Immunofluorescence analysis of autophagy-related proteins. **(A)** Colocalization of LC3 with p62. Arrows showed positive regions. **(B)** Quantitative fluorescence intensity of LC3. **(C)** Quantitative fluorescence intensity of p62. **(D)** Colocalized puncta were quantified. Data were analyzed with a one-way ANOVA followed by Dunnett’s multiple comparison test. ^∗^*P* < 0.05. Scale bar, 20 μm. Error bars = SD. The values of LC3 and p62 in the 0.5 T group were 1.637 ± 0.206 and 0.218 ± 0.026, respectively. The values of colocalized puncta in the 0.5 T group were 4.000 ± 1.414.

LC3 and p62 are essential for autophagy flux (Li et al., 2016). The ratio of LC3-II/I is considered an accurate indicator for autophagy formation. In this study, LC3 was detected by western blotting at 3, 4, and 5 days post-rTMS (Figure 1B). The ratio of LC3-II/I increased in the 0.5 T group compared with the normal group only on the fifth day (*P* < 0.01) (Figures 1C–E). Therefore, we focused on autophagy-related proteins on the fifth day after rTMS. Immunofluorescence assays showed that punctate LC3 staining (red dots) increased in the 0.5 T group compared with the normal group labeled by arrows, suggesting that rTMS could increase autophagosome formation (*P* < 0.05) (Figures 2A,B). p62, a marker of autophagy, was also detected. Western blotting and immunofluorescence assays (green fluorescence represented p62) showed that p62 expression in 0.5 T group compared with the normal group significantly decreased (*P* < 0.05) (Figures 1F,G, 2A,C). The results from both these assays were consistent, indicating that rTMS could enhance autolysosome degradation. In the merged images, LC3 and p62 colocalization (yellow dots) increased in the 0.5 T group labeled by arrows, whereas the colocalization of LC3 and p62 in other groups was not obvious (*P* < 0.05) (Figures 2A,D), further confirming that rTMS could increase autophagic flux.

### Repetitive Transcranial Magnetic Stimulation Activates Autophagy in an mTOR-Dependent Manner

**FIGURE 3:**
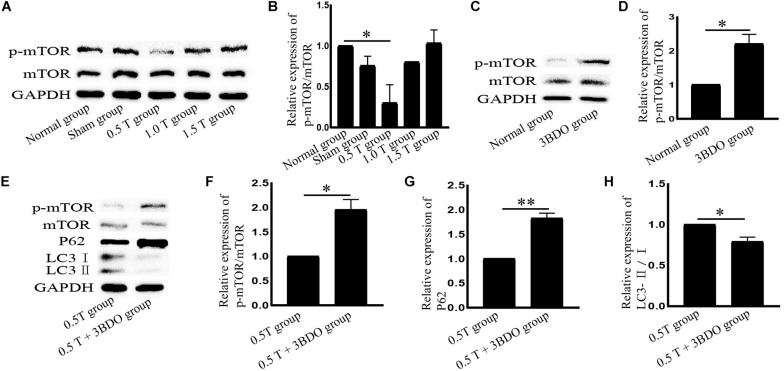
Repetitive transcranial magnetic stimulation (rTMS) mediates autophagy via mTOR. **(A)** p-mTOR and mTOR assessed by western blotting. **(B)** Quantification of western blotting for p-mTOR/mTOR. Data were analyzed with a one-way ANOVA followed by Dunnett’s multiple comparison test. ^∗^*P* < 0.05, *n* = 3. Error bars = SD. The value of p-mTOR/mTOR in the 0.5 T group was 0.301 ± 0.223. **(C)** p-mTOR and mTOR assessed by western blotting after normal group cotreatment with 3BDO. **(D)** Quantification of western blotting for p-mTOR/mTOR. Data were analyzed with a Student’s *t*-test. ^∗^*P* < 0.05, *n* = 3. Error bars = SD. The value of p-mTOR/mTOR in the MK801 group was 2.200 ± 0.283. **(E)** After cotreatment with 3BDO, the expression of p-mTOR, mTOR, LC3, and p62 was assessed by western blotting. **(F–H)** Quantification of western blotting for p-mTOR/mTOR, LC3-II/I, and p62. Data were analyzed with a Student’s *t*-test. ^∗^*P* < 0.05, ^∗∗^*P* < 0.01, *n* = 3. Error bars = SD. The values of p-mTOR/mTOR, p62, and LC3-II/I in the 0.5 T + 3BDO group were 1.950 ± 0.212, 1.825 ± 0.106, and 0.790 ± 0.057, respectively. mTOR, mammalian target of rapamycin.

Mammalian target of rapamycin signaling is a major negative regulator of autophagy. To investigate the molecular mechanisms governing the effects of rTMS, mTOR, and p-mTOR were analyzed by western blotting. In the 0.5 T group, the ratio of p-mTOR/mTOR decreased significantly (*P* < 0.05) (Figures 3A,B). These data indicated that the inhibition of mTOR signaling induced autophagy. We then used the mTOR activator 3BDO to verify these findings. The compound forms hydrogen bonds with TYR82A and ILE56A sites that represent rapamycin binding sites in FKBP1A (Sheng et al., 2017). We observed that the p-mTOR/mTOR ratio increased in cells pretreated with 3BDO (*P* < 0.05) (Figures 3C,D). Compared with those in the rTMS group, the levels of p62 and p-mTOR/mTOR increased, whereas the ratio of LC3-II/I decreased following cotreatment with 3BDO (Figures 3E–H), suggesting that autophagy was inhibited. These data suggest that mTOR inhibition is required for rTMS-induced autophagy activation.

### Repetitive Transcranial Magnetic Stimulation Upregulates ERK Expression in Bone Mesenchymal Stromal Cells

**FIGURE 4:**
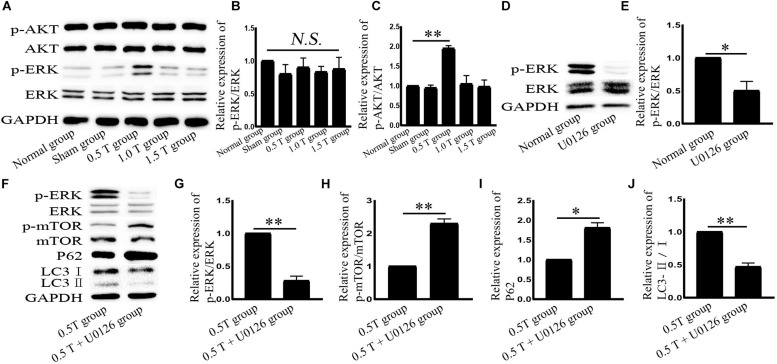
Repetitive transcranial magnetic stimulation (rTMS) activates ERK in bone mesenchymal stromal cells (BMSCs). **(A)** p-AKT, AKT, p-ERK, and ERK assessed by western blot. **(B,C)** Quantification of western blotting for p-AKT/AKT and p-ERK/ERK. Data were analyzed with a one-way ANOVA followed by Dunnett’s multiple comparison test. ^∗∗^*P* < 0.01. NS, not significant. *n* = 3. Error bars = SD. The value of p-ERK/ERK in the 0.5 T group was 1.950 ± 0.071. **(D)** p-ERK and ERK assessed by western blotting after normal group cotreatment with U0126. **(E)** Quantification of western blotting for p-ERK/ERK. Data were analyzed with a Student’s *t*-test. ^∗^*P* < 0.05, *n* = 3. Error bars = SD. The value of p-ERK/ERK in the U0126 group was 0.500 ± 0.141. **(F)** After cotreatment with U0126, ERK, p-ERK, p-mTOR, mTOR, LC3, and p62 were assessed by western blotting. **(G–J)** Quantification of western blotting for p-ERK/ERK, p-mTOR/mTOR, LC3-II/I, and p62. Data were analyzed with a Student’s *t*-test. ^∗^*P* < 0.05, ^∗∗^*P* < 0.01, *n* = 3. Error bars = SD. The values of p-ERK/ERK, p-mTOR/mTOR, p62, and LC3-II/I in the 0.5 T + U0126 treatment groups were 0.280 ± 0.071, 2.300 ± 0.141, 1.810 ± 0.127, and 0.470 ± 0.057, respectively. ERK, extracellular signal-regulated kinase; mTOR, mammalian target of rapamycin.

The two main signaling pathways upstream of mTOR are AKT and ERK (Li et al., 2016). AKT phosphorylation is used as an indicator of PI3K/AKT activation. Western blotting showed the ratio of p-AKT/AKT (Ser473) did not significantly change in the 0.5 T group (*P* > 0.05) (Figures 4A,B). These results suggest that rTMS induces autophagy through a mechanism independent of PI3K/AKT. ERK is a major negative regulator of mTOR. In this study, rTMS could upregulate the ratio of p-ERK/ERK in the 0.5 T group (*P* < 0.01) (Figures 4A,C). The data indicated that ERK signaling regulates rTMS activation. An ERK inhibitor (U0126) was used to confirm these findings. We observed a decrease in p-ERK/ERK in cells pretreated with U0126 (*P* < 0.05) (Figures 4D,E). We also found a reversal of p-mTOR/mTOR, p62, and LC3-II/I levels in response to rTMS following cotreatment with U0126 (Figures 4F–J). These data indicated that rTMS inhibited mTOR via the ERK pathway.

### Repetitive Transcranial Magnetic Stimulation Upregulates NMDAR1 Expression in Bone Mesenchymal Stromal Cells

**FIGURE 5:**
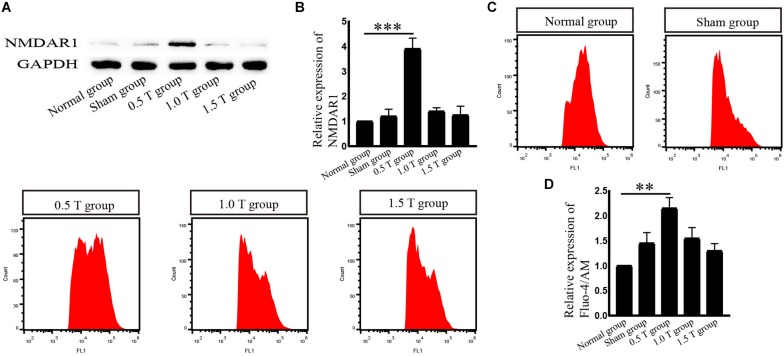
Repetitive transcranial magnetic stimulation (rTMS) mediates the levels of NMDAR1 and intracellular calcium (Ca^2+^) in bone mesenchymal stromal cells (BMSCs). **(A)** NMDAR1 assessed by western blotting. **(B)** Quantification of western blotting for NMDAR1. Data were analyzed with a one-way ANOVA followed by Dunnett’s multiple comparison test. ^∗∗∗^*P* < 0.001, *n* = 3. Error bars = SD. The value of NMDAR in the 0.5 T group was 3.900 ± 0.424. **(C)** Detection of Ca^2+^ levels by Fura-4/AM after rTMS. **(D)** Quantitative fluorescence intensity of Ca^2+^ levels. Data were analyzed with a one-way ANOVA followed by Dunnett’s multiple comparison test. ^∗∗^*P* < 0.01, *n* = 3. Error bars = SD. The value of Ca^2+^ in the 0.5 T group was 2.150 ± 0.212.

**FIGURE 6:**
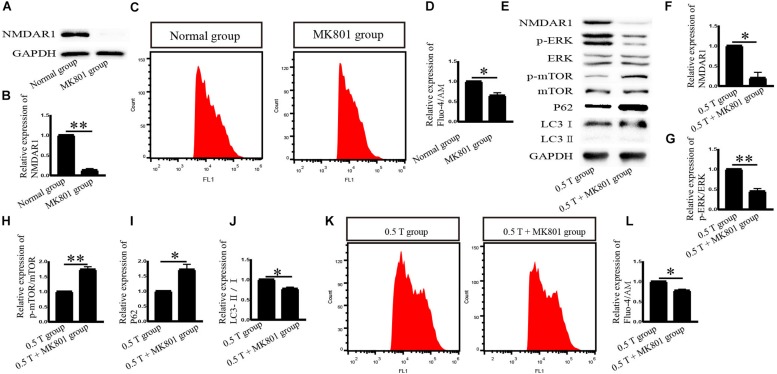
Effects of MK801 on NMDAR1 after incubation with rTMS in bone mesenchymal stromal cells (BMSCs). **(A)** NMDAR1 assessed by western blotting after normal group cotreatment with MK801. **(B)** Quantification of western blotting for NMDAR1. Data were analyzed with a Student’s *t*-test. ^∗∗^*P* < 0.01, *n* = 3. Error bars = SD. The value of NMDAR1 in the MK801 group was 0.130 ± 0.042. **(C)** Detection of Ca^2+^ levels after normal group cotreatment with MK801. **(D)** Quantitative fluorescence intensity of Ca^2+^ levels. Data were analyzed with a Student’s *t*-test. ^∗^*P* < 0.05, *n* = 3. Error bars = SD. The value of Ca^2+^ in the MK801 group was 0.650 ± 0.071. **(E)** Cells were pretreated with MK801 (10 μM) for 1 h and incubated with rTMS. NMDAR1, ERK, p-ERK, p-mTOR, mTOR, LC3, and p62 were assessed by western blotting. **(F–J)** Quantification of western blotting for NMDAR1, p-ERK/ERK, p-mTOR/mTOR, LC3-II/I, and p62. Data were analyzed with a Student’s *t*-test. ^∗^*P* < 0.05, ^∗∗^*P* < 0.01, *n* = 3. Error bars = SD. The values of NMDAR1, p-ERK/ERK, p-mTOR/mTOR, p62, and LC3-II/I in the 0.5 T + MK801 group were 0.200 ± 0.141, 0.450 ± 0.071, 1.735 ± 0.092, 1.715 ± 0.177, and 0.770 ± 0.042, respectively. **(K)** Detection of intracellular Ca^2+^ levels after cotreatment with MK801. **(L)** Quantitative fluorescence intensity of Ca^2+^ levels assessed by Fura-4/AM. Data were analyzed with a Student’s *t*-test. ^∗^*P* < 0.05, *n* = 3. Error bars = SD. The value of Ca^2+^ in the 0.5 T + MK801 group was 0.775 ± 0.035. ERK, extracellular signal-regulated kinase; mTOR, mammalian target of rapamycin.

NMDA receptor subtype of glutamate-gated ion channels has high Ca^2+^ permeability, which is mediated by rTMS (Baek et al., 2018b). In this study, the NMDAR subtype of glutamate-gated ion channels was investigated. Western blotting analysis showed that NMDAR1 increased in the 0.5 T group (*P* < 0.001) (Figures 5A,B). Intracellular calcium (Ca^2+^) was detected by flow cytometry after incubation with Fura-4/AM 30 min. rTMS dramatically enhanced intracellular Ca^2+^ levels compared with those in the normal group (*P* < 0.01) (Figures 5C,D). These results demonstrate that rTMS could upregulate the expression of NMDAR1 and increase the levels of intracellular Ca^2+^. Whether the NMDAR–Ca^2+^ pathway is required for the rTMS regulation autophagy is also assessed. The NMDAR antagonist (MK801) was used, which occupied the NMDAR binding site of the Ca^2+^ channel and prevented Ca^2+^ ions from entering the cell through NMDAR channels. When pretreated with MK801, western blotting showed that the levels of NMDAR1 decreased (*P* < 0.01) (Figures 6A,B), and the percentage of cells stained with Fura-4/AM decreased by ∼35% (*P* < 0.05) (Figures 6C,D). Compared with that in the rTMS group, cotreatment with MK801 decreased the expression of NMDAR1, p-ERK/ERK, and LC3-II/I and increased the levels of p-mTOR/mTOR and p62. The percentage of cells stained by Fura-4/AM was also decreased by ∼23% (Figures 6E–L). Taken together, the NMDAR–Ca^2+^ pathway is required for rTMS regulation autophagy.

### Repetitive Transcranial Magnetic Stimulation Does Not Affect the Stemness of Bone Mesenchymal Stromal Cells

**FIGURE 7:**
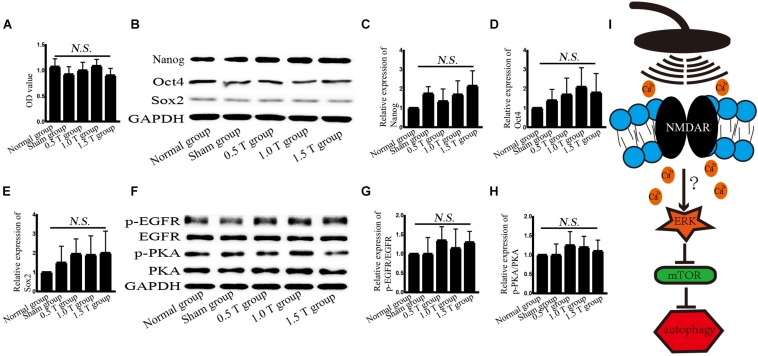
Repetitive transcranial magnetic stimulation (rTMS) does not affect the stemness of bone mesenchymal stromal cells (BMSCs). **(A)** Cell viability was assessed by Cell Counting Kit-8 assays. *n* = 3–5. All data were analyzed with a one-way ANOVA followed by Dunnett’s multiple comparison test. NS, not significant. **(B)** Nanog, Oct4, and Sox2 were assessed by western blotting. **(C–E)** Quantification of western blotting for Nanog, Oct4, and Sox2. Data were analyzed with a one-way ANOVA followed by Dunnett’s multiple comparison test. NS, not significant. *n* = 3. Error bar = SD. **(F)** phosphorylated epidermal growth factor receptor (p-EGFR), EGFR, p-PKA, and PKA were assessed by western blotting. **(G,H)** Quantification of western blotting for p-EGFR/EGFR and p-PKA/PKA. Data were analyzed with a one-way ANOVA followed by Dunnett’s multiple comparison test. NS, not significant. *n* = 3. Error bar = SD. **(I)** Schematic diagram of the autophagy pathway induced by rTMS in BMSCs.

To assess the stemness of BMSCs, cell viability is detected. After incubation with CCK-8 for 1.5 h, cell viability was detected at once. Treatment with rTMS for 5 days did not significantly affect the viability of BMSC cells (*P* > 0.05) (Figure 7A). This excluded the non-specific impact of rTMS on autophagy that may be related to cell damage or death. The stemness-related proteins Nanog, Oct4, and Sox2 were detected by western blotting (Figure 7B). The results showed no statistically significant differences (*P* > 0.05) (Figures 7C–E).

## Discussion

The results of this study demonstrated that rTMS could regulate autophagy *in vitro*. Furthermore, NMDAR–Ca^2+^–ERK–mTOR signaling was identified as critical for autophagy induction by rTMS in BMSCs.

High-frequency rTMS is used in clinic and can temporarily modulate cortical activity. However, previous studies did not clarify the effects of rTMS on the regulation of autophagy and its associated molecular changes *in vitro*. The purpose of this study was to provide experimental evidence that rTMS mediates BMSCs. The primary advantage of these *in vitro* studies was that additional complications of penetration through the skin and bone were removed. As rTMS acts directly on the tissue of interest, the procedure was cost-effective (Hellmann et al., 2012). High-frequency rTMS (10 Hz) increases growth factor stimulation by activating AKT and ERK signaling cascades in neuroblastoma cells (Baek et al., 2018a). The levels of AKT do not increase in this study, and autophagy is induced after 5 days of rTMS treatment. These results are related to the rTMS parameters and implicated an AKT independent role of autophagy induction.

No studies have explored the mechanisms of BMSCs by rTMS. High-frequency rTMS (20 Hz) has been shown to promote neural stem cell proliferation, but no such effects on BMSCs were observed in this study (Luo et al., 2017). These discrepancies may be related not only to the rTMS parameters but also the stemness of the BMSCs. In this study, BMSC was used from passages 3 to 7, and the stemness of the BMSCs was detected. No changes in the expression of Nanog, Oct4, and Sox2 were observed, all of which may be influenced by autophagy, but the stemness of BMSCs is affected by many factors, including microRNA-specific expression profiles, DNA methylation, and histone modifications, and these factors may have influenced the findings.

In our experiments, the effects of different intensities for autophagy formation and autophagy-related proteins were investigated. NMDAR–Ca^2+^–ERK–mTOR signaling was identified as involved. NMDARs have two subunits: NR1 and NR2. As a functional subunit, NR1 has the basic functional characteristics of NMDARs, whereas NR1 activation plays an important role in spatial learning and memory (Huang et al., 2015). Western blotting showed that the expression of NMDA1 increased in response to rTMS in the 0.5 T group (Figures 5A,B). NMDA could mediate Ca^2+^ flux. The Ca^2+^ increased in rTMS group and reduced in the presence of the NMDAR antagonist (MK801) (Figures 5C,D, 6C,D). Therefore, we speculated that rTMS activation NMDAR signaling promoted the flux of Ca^2+^. Intracellular Ca^2+^ elevations were required for the activation of EGFR, and it was reported that 50 Hz at 0.1, 0.2, or 0.4 mT for 15 min could significantly increase the levels of p-EGFR in Chinese hamster lung cells (Sun et al., 2008). In this study, the ratio of p-EGFR/EGFR did not significantly change in the 0.5 T group (*P* > 0.05) (Figures 7F,G). PKA was an upstream signaling protein of ERK (Zhao et al., 2019). But the levels of p-PKA/PKA did not change in this study (*P* > 0.05) (Figures 7F,H). rTMS could upregulate the expression of p-ERK/ERK, and LC3-II/I and U0126 reversed these effects, implicating ERK-mediated signaling in the induction of autophagy. mTOR serves as a major regulator of autophagy. Our results demonstrated that rTMS repressed the phosphorylation mTOR and that the mTOR activator 3BDO decreased the autophagy induction. The data demonstrated that mTOR signaling was closely related to the autophagy induction observed in response to rTMS. rTMS also did not increase the expression of p-AKT, further implicating ERK rather than AKT as critical during autophagy induced by rTMS.

Autophagy is a lysosomal degradation pathway that widely occurs in all eukaryotic cells, playing an important role in cell metabolism. Autophagy as a therapeutic intervention can treat CNS diseases, such as Parkinson’s and Alzheimer’s. Autophagy regulates the balance between cell survival and death. Increasing autophagy flux had been shown to offer neuroprotection (Lipinski et al., 2015). Autophagy flux refers to the process of autophagosome production and the degradation of intracellular proteins/organelles after their combination with lysosomes to regulate intracellular homeostasis. Enhanced levels of LC3-II or the ratio of LC3-II/I do not alter autophagy flux but act as measurements of autophagosome accumulation. Hence, we examined the formation and degradation of autolysosomes using p62 as a marker, to ensure the state of autophagic flux. Our data revealed that the ratio of LC3-II/I increased and p62 decreased, suggesting that autophagy flux occurred on the fifth day of rTMS (Figures 1E,G, 2B,C). The results of immunofluorescence assays and western blotting were consistent. No changes in LC3-II/I and p62 were observed in 1.0 T and 1.5 T groups. This revealed for the first time the mechanisms by which rTMS could induce autophagy *in vitro* (summarized in Figure 7I).

In summary, the molecular responses of BMSCs to rTMS have been characterized. In general, autophagy flux is protective and induced by rTMS via the NMDAR–Ca^2+^–ERK–mTOR signaling in BMSCs. This study improves our understanding of the mechanisms by which rTMS affects BMSCs.

## Data Availability Statement

The datasets generated for this study are available on request to the corresponding author.

## Author Contributions

XW and XZ prepared the manuscript and performed the experiments. JB, ZC, JT, XG, and JN assisted in conducting many of the experiments. QF, YL, and MS conceived the idea for the project and contributed to the experimental design. All authors read and approved the final manuscript.

## Conflict of Interest

The authors declare that the research was conducted in the absence of any commercial or financial relationships that could be construed as a potential conflict of interest.

## References

[B1] Abe K. Yamashita T. Takizawa S. Kuroda S. Kinouchi H. Kawahara N. (2012). Stem cell therapy for cerebral ischemia: from basic science to clinical applications. *J. Cereb. Blood Flow Metab.* 32 1317–1331. 10.1038/jcbfm.2011.187 22252239 PMC3390814

[B2] Ao L. Y. Li W. T. Zhou L. Yan Y. Y. Ye A. Q. Liang B. W. (2019). Therapeutic effects of JLX-001 on ischemic stroke by inducing autophagy via AMPK-ULK1 signaling pathway in rats. *Brain Res. Bull.* 153 162–170. 10.1016/j.brainresbull.2019.08.017 31472184

[B3] Baek A. Kim J. H. Pyo S. Jung J. H. Park E. J. Kim S. H. (2018a). The differential effects of repetitive magnetic stimulation in an in vitro neuronal model of ischemia/reperfusion injury. *Front. Neurol.* 9:50. 10.3389/fneur.2018.00050 29487560 PMC5816832

[B4] Baek A. Park E. J. Kim S. Y. Nam B. G. Kim J. H. Jun S. W. (2018b). High-frequency repetitive magnetic stimulation enhances the expression of brain-derived neurotrophic factor through activation of Ca(2+)-calmodulin-dependent protein kinase ii-camp-response element-binding protein pathway. *Front. Neurol.* 9:285. 10.3389/fneur.2018.00285 29867712 PMC5949612

[B5] Fang E. F. Hou Y. Palikaras K. Adriaanse B. A. Kerr J. S. Yang B. (2019). Mitophagy inhibits amyloid-beta and tau pathology and reverses cognitive deficits in models of Alzheimer’s disease. *Nat. Neurosci.* 22 401–412. 30742114 10.1038/s41593-018-0332-9 PMC6693625

[B6] Hellmann J. Juttner R. Roth C. Bajbouj M. Kirste I. Heuser I. (2012). Repetitive magnetic stimulation of human-derived neuron-like cells activates cAMP-CREB pathway. *Eur. Arch. Psychiatry Clin. Neurosci.* 262 87–91. 21562895 10.1007/s00406-011-0217-3

[B7] Huang J. Hult E. F. Marchal E. Tobe S. S. (2015). Identification and characterization of the NMDA receptor and its role in regulating reproduction in the cockroach diploptera punctata. *J. Exp. Biol.* 218(Pt 7), 983–990. 10.1242/jeb.115154 25657209

[B8] Li X. Cen Y. Cai Y. Liu T. Liu H. Cao G. (2016). TLR9-ERK-mTOR signaling is critical for autophagic cell death induced by CpG oligodeoxynucleotide 107 combined with irradiation in glioma cells. *Sci. Rep.* 6:27104. 10.1038/srep27104 27251306 PMC4890034

[B9] Lipinski M. M. Wu J. Faden A. I. Sarkar C. (2015). Function and mechanisms of autophagy in brain and spinal cord trauma. *Antioxid. Redox Signal.* 23 565–577. 10.1089/ars.2015.6306 25808205 PMC4545370

[B10] Luo J. Zheng H. Zhang L. Zhang Q. Li L. Pei Z. (2017). High-frequency repetitive transcranial magnetic stimulation (rTMS) improves functional recovery by enhancing neurogenesis and activating BDNF/TrkB signaling in ischemic rats. *Int. J. Mol. Sci.* 18:E455. 10.3390/ijms18020455 28230741 PMC5343989

[B11] Matheson N. A. Shemmell J. B. De Ridder D. Reynolds J. N. (2016). Understanding the effects of repetitive transcranial magnetic stimulation on neuronal circuits. *Front. Neural Circuits* 10:67. 10.3389/fncir.2016.00067 27601980 PMC4993761

[B12] Rabey J. M. Dobronevsky E. (2016). Repetitive transcranial magnetic stimulation (rTMS) combined with cognitive training is a safe and effective modality for the treatment of Alzheimer’s disease: clinical experience. *J. Neural Transm.* 123 1449–1455. 27631152 10.1007/s00702-016-1606-6

[B13] Sheng Y. L. Chen X. Hou X. O. Yuan X. Yuan B. S. Yuan Y. Q. (2017). Urate promotes SNCA/alpha-synuclein clearance via regulating mTOR-dependent macroautophagy. *Exp. Neurol.* 297 138–147. 10.1016/j.expneurol.2017.08.007 28821398

[B14] Shu X. Chen S. Chai G. Sheng X. Jia J. Zhu X. (2018). Neural modulation by repetitive transcranial magnetic stimulation (rTMS) for BCI enhancement in stroke patients. *Conf. Proc. IEEE Eng. Med. Biol. Soc.* 2018 2272–2275. 10.1109/embc.2018.8512860 30440859

[B15] Su L. Y. Li H. Lv L. Feng Y. M. Li G. D. Luo R. (2015). Melatonin attenuates MPTP-induced neurotoxicity via preventing CDK5-mediated autophagy and SNCA/alpha-synuclein aggregation. *Autophagy* 11 1745–1759. 10.1080/15548627.2015.1082020 26292069 PMC4824603

[B16] Sun W. Gan Y. Fu Y. Lu D. Chiang H. (2008). An incoherent magnetic field inhibited EGF receptor clustering and phosphorylation induced by a 50-Hz magnetic field in cultured FL cells. *Cell. Physiol. Biochem.* 22 507–514. 10.1159/000185524 19088432

[B17] To W. T. De Ridder D. Hart J. Jr. Vanneste S. (2018). Changing brain networks through non-invasive neuromodulation. *Front. Hum. Neurosci.* 12:128. 10.3389/fnhum.2018.00128 29706876 PMC5908883

[B18] Towers C. G. Thorburn A. (2016). Therapeutic targeting of autophagy. *EBioMedicine* 14 15–23. 10.1016/j.ebiom.2016.10.034 28029600 PMC5161418

[B19] Wang F. Zhang C. Hou S. Geng X. (2018). Synergistic effects of mesenchymal stem cell transplantation and repetitive transcranial magnetic stimulation on promoting autophagy and synaptic plasticity in vascular dementia. *J. Gerontol. A Biol. Sci. Med. Sci.* 74 1341–1350. 10.1093/gerona/gly221 30256913

[B20] Wu X. Fleming A. Ricketts T. Pavel M. Virgin H. Menzies F. M. (2016). Autophagy regulates notch degradation and modulates stem cell development and neurogenesis. *Nat. Commun.* 7:10533. 10.1038/ncomms10533 26837467 PMC4742842

[B21] Yang C. Guo Z. Peng H. Xing G. Chen H. McClure M. A. (2018). Repetitive transcranial magnetic stimulation therapy for motor recovery in Parkinson’s disease: a meta-analysis. *Brain Behav.* 8:e01132. 10.1002/brb3.1132 30264518 PMC6236247

[B22] Zhang M. Tao W. Yuan Z. Liu Y. (2017). Mst-1 deficiency promotes post-traumatic spinal motor neuron survival via enhancement of autophagy flux. *J. Neurochem.* 143 244–256. 10.1111/jnc.14154 28833175

[B23] Zhao Q. Ma Y. M. Jing L. Zheng T. X. Jiang H. F. Li P. A. (2019). Coenzyme Q10 protects astrocytes from ultraviolet B-induced damage through inhibition of ERK 1/2 pathway overexpression. *Neurochem. Res.* 44 1755–1763. 31093903 10.1007/s11064-019-02812-6

